# Acanthosis Nigricans in a Patient with Lung Cancer: A Case Report

**DOI:** 10.1155/2010/412159

**Published:** 2010-08-09

**Authors:** Duru Serap, Sever Özlem, Yüceege Melike, Dilli Alper, Albayrak Aynur, Arıkök Ata Türker, Sadik Ardıç

**Affiliations:** ^1^Dışkapı Yıldırım Bayazıt Education and Reserch Hospital, Chest Diseases Clinic, 06200 Ankara, Turkey; ^2^Dışkapı Yıldırım Bayazıt Education and Reserch Hospital, Radiology, 06200 Ankara, Turkey; ^3^Dışkapı Yıldırım Bayazıt Education and Reserch Hospital, 2nd Pathology Clinic, 06200 Ankara, Turkey

## Abstract

Some skin lesions may accompany malignancies. Acanthosis nigricans, one such lesion, is a paraneoplastic dermatosis characterized by hyperpigmented and velvety verrucose plaques observed as symetric eruptions. With this report, we aim to present a rare case of concomitant lung cancer and acanthosis nigricans. Malignant acanthosis nigricans is most commonly associated with intra-abdominal malignancies. A 65-year-old patient who had hyperpigmented, hypertrophic and symmetric verrucose lesions at the flexor surfaces of the lower and upper extremities, face, palms and the axillary region. Thoracic computed tomography demonstrated a hypodense mass lesion with a dimension of 5 × 5.5 cm at the center of basal segment bronchi of the left pulmonary lobe. Fiberoptic bronchoscopy showed that the access to the lower left lobe was almost completely obstructed by the endobronchial lesion. The result of the histopathologic examination of the endobronchial tissue biopsy was reported as non-small cell (adenocarcinoma) lung cancer. Result of the histopathologic analysis of the punch biopsy of the skin lesions was reported as acanthosis nigricans .There are no pathognomonic dermatological findings for lung cancer. In conclusion, there are skin lesions that accompany lung cancer and we believe that these should be considered for differential diagnosis.

## 1. Introduction

Some skin lesions may accompany malignancies. Acanthosis nigricans, one such lesion, is a paraneoplastic dermatosis characterized by hyperpigmented and velvety verrucose plaques observed as symmetric eruptions. 

 It is often localized on flexor surfaces such as the axilla, posterior neck fold, anterior umbilical, and popliteal and inguinal areas where skin folds. It may be idiopathic but may also be associated with endocrinal disorders, malignancies, medicines, and genetic syndromes. Besides, acanthosis nigricans may also present autosomal dominant involvement [1–3].

Malignant acanthosis nigricans is most commonly associated with intra-abdominal malignancies. There are very few reports in the literature of malignant acanthosis nigricans associated with lung cancer. 

With this report, we aim to present a rare case of concomitant lung cancer and acanthosis nigricans.

## 2. Case Presentation

A 65-year-old male patient who presented to our clinic had the complaints of cough and phlegm for the past 10 years which particularly exacerbated in winter and shortness of breath exacerbating with effort for the past one year. An informed consent was taken from the patient. Patient had no comorbid disease including diabetes mellitus.

The patient also had dark-colored lesions that he first observed one month ago and spread to the body thereafter, and the additional complaints of worsened shortness of breath and weight loss. His history involved smoking, 60 boxes/year. His familial history had no relevant findings. Physical examination revealed the following values: BP: 120/80 mmHg, RR: 16/min, and temperature 36.5°C. The patient had body weight: 64 kg and body length: 170 cm, BMI: 22.14 kg/m².

The patient had hyperpigmented, hypertrophic and symmetric verrucose lesions at the flexor surfaces of the lower and upper extremities, face, palms, and the axillary region. An examination of the respiratory tract with auscultation revealed bilateral infrequent expiratory rhoncus. Findings of other examinations were normal. Laboratory findings showed an ESR value of 64 m/h, fasting blood glucose as 74 mg/dl. Compensated respiratory acidosis was observed during blood gas analysis. Other laboratory findings were within normal ranges. 

**Figure 1:**
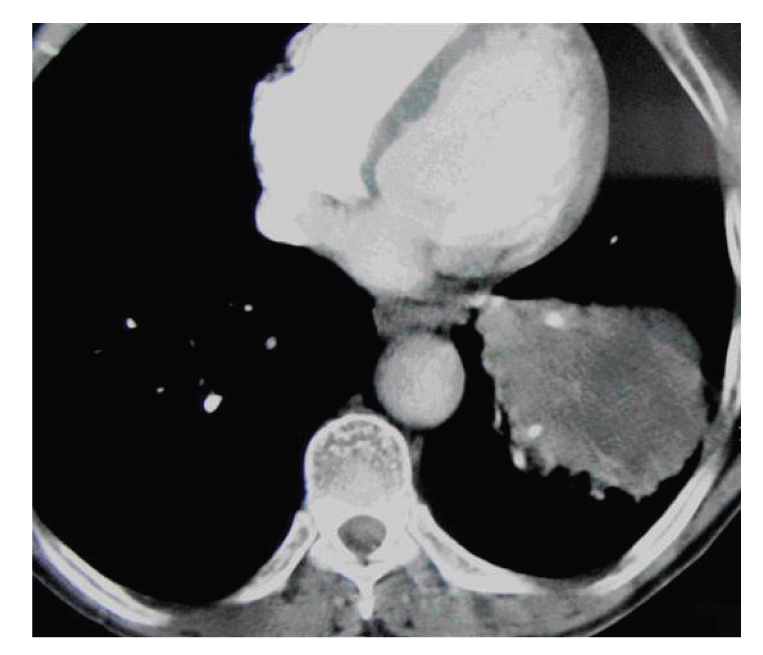
Thoracic computed tomography (a hypodense mass lesion at the left pulmonary lobe).

Thoracic computed tomography demonstrated a hypodense mass lesion with a dimension of 5 × 5.5 cm at the center of basal segment bronchi of the left pulmonary lobe, which did not have clear borders distinguishable from the peripheral consolidation region (Figure 1). 

Fiberoptic bronchoscopy showed that the access to the lower left lobe was almost completely obstructed by the endobronchial lesion. 

**Figure 3:**
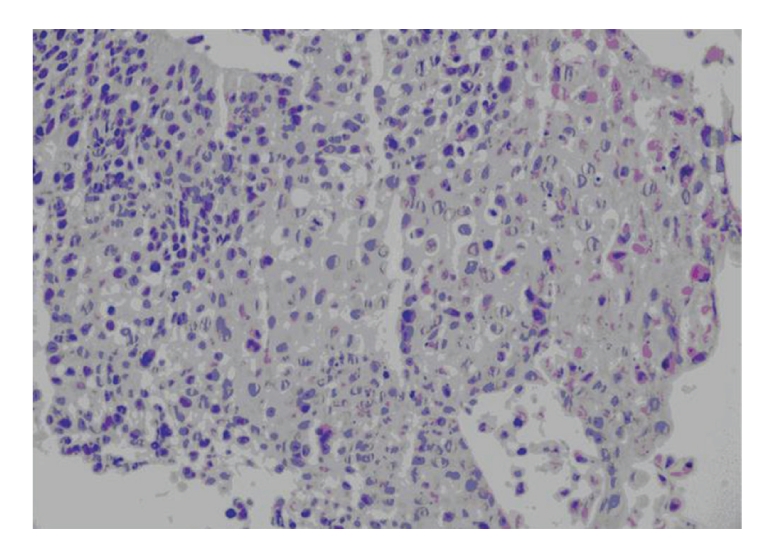
Coarse hyperchromatic atypical cell cluster with an average-width eosinophilic cytoplasm with evident nucleolus (H&E, x40 obj).

Abdominal tomography, esophagoduodenoscopic, colonoscopic examination revealed no malignancies, only erosive gastritis was found. Primary lesion was considered as the primary tumor as there was no malignancy at any organ. The result of the histopathologic examination of the endobronchial tissue biopsy was reported as nonsmall cell (adenocarcinoma) lung cancer (Figure 3).

**Figure 4:**
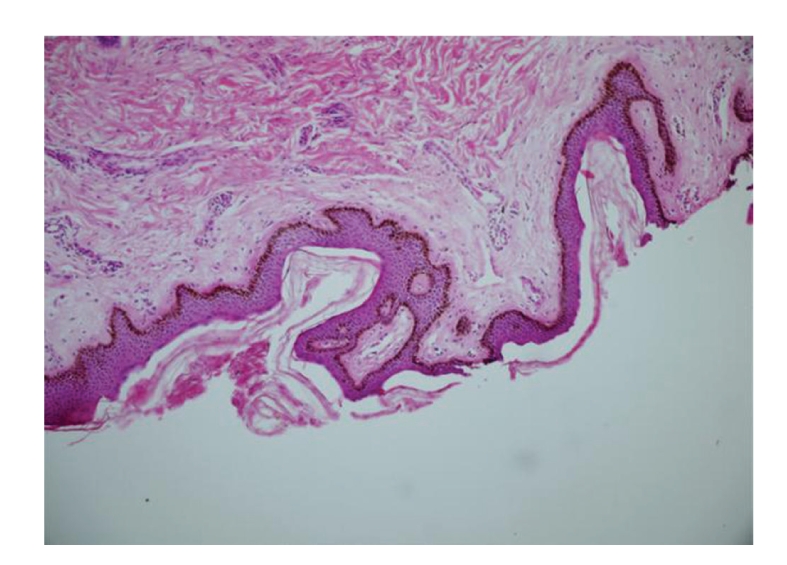
Hyperkeratosis, papillomatous mild acanthosis in the epidermis and hyperpigmentation at the basal layer (H& E, x20 obj).

Result of the histopathologic analysis of the punch biopsy of the skin lesions was reported as acanthosis nigricans (Figure 4). 

## 3. Discussion

There are no pathognomonic dermatological findings for lung cancer. However, some skin lesions should suggest lung cancer in differential diagnosis. Acanthosis nigricans is one such lesion. Acanthosis nigricans may both be benign or malign. Its benign form may be observed in association with some endocrinal disorders, obesity, some genetic syndromes, and drug use (nicotinic acid, estrogen, corticosteroids) [4, 5]. We could not manage to find the actual percentage for “*idiopathic acanthosis nigricans*” from the literatures, but we consider that the most of the cases can be associated with a disease. 

 Malign acanthosis nigricans accounts for 20% of all acanthosis nigricans cases. It develops as a result of expression of the transforming growth factor alpha (TGF alpha) of tumor cells, melanocyte stimulating hormone alpha, and peptides causing cellular proliferation including insulin-like growth factor 1 [6].

Of the malignancies accompanying malign acanthosis nigricans in the adult population, 45–69% are gastric adenocarcinomas. Malign acanthosis nigricans is observed with a frequency of 70–92% of all abdominal neoplasms [7–9]. approximately, 9% of all MAN cases are related with extra-abdominal malignancies: Endometrium [10] lymphoma [11], mycosis fungoides, melanoma, and sarcomas [12]. These are all presented as case reports.

After diagnosing as acanthosis nigricans, benign diseases were investigated (no diabetes, obesity, drug use such as nicotinic acid, estrogen, or corticosteroid). After that, possibility malign disease was thougt. The most common form of malignancy with MAN, the abdominal malignancy was investigated by abdominal tomography, esophagoduodenoscopy, colonoscopy, and rectoscopy which were all normal. The edoscopic investigations should be done in every patient with MAN. They all revealed normal findings in this patient. Brain computerised tomography and bone scintigraphy revealed no lesions. If there were no lung cancer in our case, the other rare malignancies with MAN listed above would have been searched. 

First described by Pollitzer in 1891, acanthosis nigricans is a key indicator of insulin resistance independent from the body mass index [13–15]. It has an increasing incidence in obese children and adults. It may be seen together with other skin disorders in patients with insulin resistance and uncontrolled diabetes [16, 17]. The patient had no diabetic disease. 

Co-occurrence of acanthosis nigricans and lung cancers is rare. The association with bronchial carcinoma was first reported in 1909 [18]. Curth et al. [19] extensively reviewed the association of pulmonary neoplasms and acanthosis nigricans. To date, eight cases of adenocarcinoma of the lung and five cases of squamous carcinoma of the lung have been reported in association with acanthosis nigricans [20]. A patient with bronchoalveoler carsinom is reported [21]. And only few cases with pulmonary malignancy with acanthosis nigricans are reported in the literature [22, 23]. It alone may predict malignancy or may develop with other signs or occur later. Tumors are often in the advanced phase even when malign acanthosis nigricans is diagnosed simultaneously with the tumor. Since the patient had no radiography at the early phase and his recent radiography showed a large mass in the lung, we believe that the skin lesions developed later. Treatment of malign acanthosis nigricans depends on the treatment of the underlying malignancy. Skin lesions usually regress as treatment success is achieved. 

No masses were detected during the scans of the other organs. Being assessed as advanced phase nonsmall cell lung cancer, the patient was examined by the oncology department. Chemotherapy and radiotherapy were initiated and regressions were noted in the patient's skin lesions, consistent with the literature. The patient refused to go through thorasic surgery and died three months after presentation due to respiratory failure.

In conclusion, there are skin lesions that accompany lung cancer and we believe that these should be considered for differential diagnosis.

## Figures and Tables

**Figure 2:**
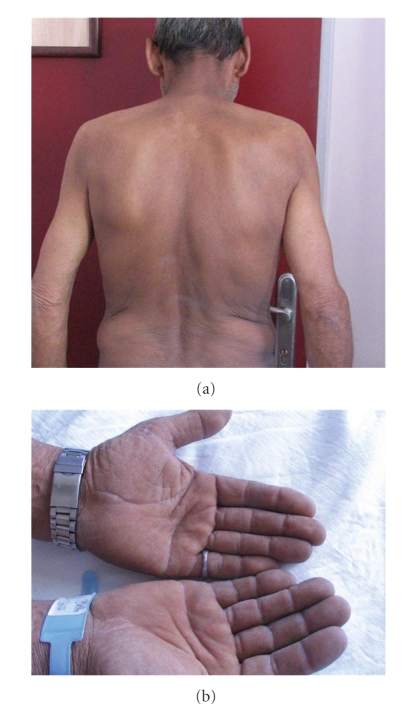
Hyperpigmentation in the back and hands.
